# Deep Coral Oases in the South Tyrrhenian Sea

**DOI:** 10.1371/journal.pone.0049870

**Published:** 2012-11-21

**Authors:** Marzia Bo, Simonepietro Canese, Costanza Spaggiari, Antonio Pusceddu, Marco Bertolino, Michela Angiolillo, Michela Giusti, Maria Filomena Loreto, Eva Salvati, Silvestro Greco, Giorgio Bavestrello

**Affiliations:** 1 Dipartimento per lo Studio del Territorio e delle sue Risorse, Università degli Studi di Genova, Genova, Italy; 2 Istituto Superiore per la Protezione e la Ricerca Ambientale, Roma, Italy; 3 Dipartimento di Scienze della Vita e dell’Ambiente, Università Politecnica delle Marche, Ancona, Italy; 4 Istituto Nazionale di Oceanografia e di Geofisica Sperimentale di Trieste, Trieste, Italy; Istituto di Scienze Marine - CNR U.O.S. Bologna, Bologna, Italy; Leibniz Center for Tropical Marine Ecology, Germany

## Abstract

A Mediterranean “roche du large” ecosystem, represented by four rocky shoals, located a few miles apart on a muddy bottom at 70–130 m depth in the gulf of St. Eufemia (Calabria, South Tyrrhenian Sea), was studied by means of Remotely Operated Vehicle (ROV) photo imaging. The shoals host highly diversified coral communities, mainly composed of arborescent colonies of gorgonians (*Callogorgia verticillata*, *Paramuricea clavata*, *Paramuricea macrospina*, *Bebryce mollis*, *Villogorgia bebrycoides*, *Corallium rubrum*, and *Leptogorgia sarmentosa*), and antipatharians (*Antipathella subpinnata*, *Antipathes dichotoma* and *Parantipathes larix*). The coral colonies reach high densities (up to ca. 17 colonies m^−2^) and large sizes, such as the over 1.5 m wide antipatharian colonies. We hypothesized that the abundance and composition of the coral assemblages differed significantly among the rocky shoals and with respect to the surrounding soft bottoms. Various environmental variables were tested as possible explanatory factors of the observed differences. Moreover, due to their off-coast localization, we report here that these unique ecosystems are potentially subjected to a strong pressure from the local fishing activities, which were tentatively characterized. The recorded coral β-diversity among the shoals supports the hypothesis that these habitats behave like small oases of hard substrata interspersed in a muddy bottom. Because of their intrinsic beauty and rarity and their biological and ecological value, we stress the need of specific actions aimed at the urgent protection of these oases of biodiversity.

## Introduction

Mediterranean Sea biodiversity has been deeply studied over the past centuries; nevertheless, large portions of it, especially in the deep realm, still remain largely unknown [1], [2], [3], [4]. With respect to the traditional bionomic characterization of the Mediterranean Sea [5], only few quantitative data currently exist dealing with the distribution of the megafaunal “roche du large” benthic communities. These represent poorly studied biocoenoses of the deep circa-littoral rocky bottoms mainly composed of sponges, anthozoans, bryozoans and brachiopods and generally lacking the macroscopic algal component [5], [6]. Found near the border of the continental shelf, these biocoenoses, dwelling to 250 m depth, usually occupy rough bottoms where current and turbidity are reasonably important [5].

In these habitats, the progressively lower light conditions have a profound influence on the biocoenoses composition mostly because of reduced primary production, but this zone continues to be characterized by high biodiversity and biomass levels, at times comparable to those found in shallow waters [4], [7], [8]. Such biological and ecological richness is influenced by several constraining factors, related not only to environmental and biological cues but also to human activities, since most of these habitats are a potential fishing ground [4], [8].

The biodiversity of the deep Mediterranean habitats has been investigated for a long time using invasive and often blind sampling techniques [for example 9, 10, 11], whereas direct and non-destructive observations, i.e. with the use of Remotely Operated Vehicles (ROVs) or technical diving, are much more recent. These studies, carried out using direct observational techniques, revealed the great species richness of the Mediterranean deep rocky circa-littoral environments which are characterized by a large variety and abundance of sponges and corals [4], [5], [7], [8], [12], [13], [14], [15], [16], [17], [18], [19], [20], [21], [22]. Among these biocoenoses, large benthic cnidarians can play an important ecological role; they are considered ecosystem engineers, creating complex three-dimensional habitats, sustaining high biodiversity levels and promoting high levels of functioning in both epi-benthic and proximal interstitial surroundings [7], [23]. These organisms represent a colonizable substrate [8], create refuges for numerous other species [12] and may represent a nursery area for fish [24]. The rocky habitats hosting biocoenoses dominated by cnidarians assemblages are key environments when surrounded by soft bottoms, because they concentrate larval settling and sinking of organic material, thus favouring the development of benthic communities also in their vicinity [7].

**Figure 1:**
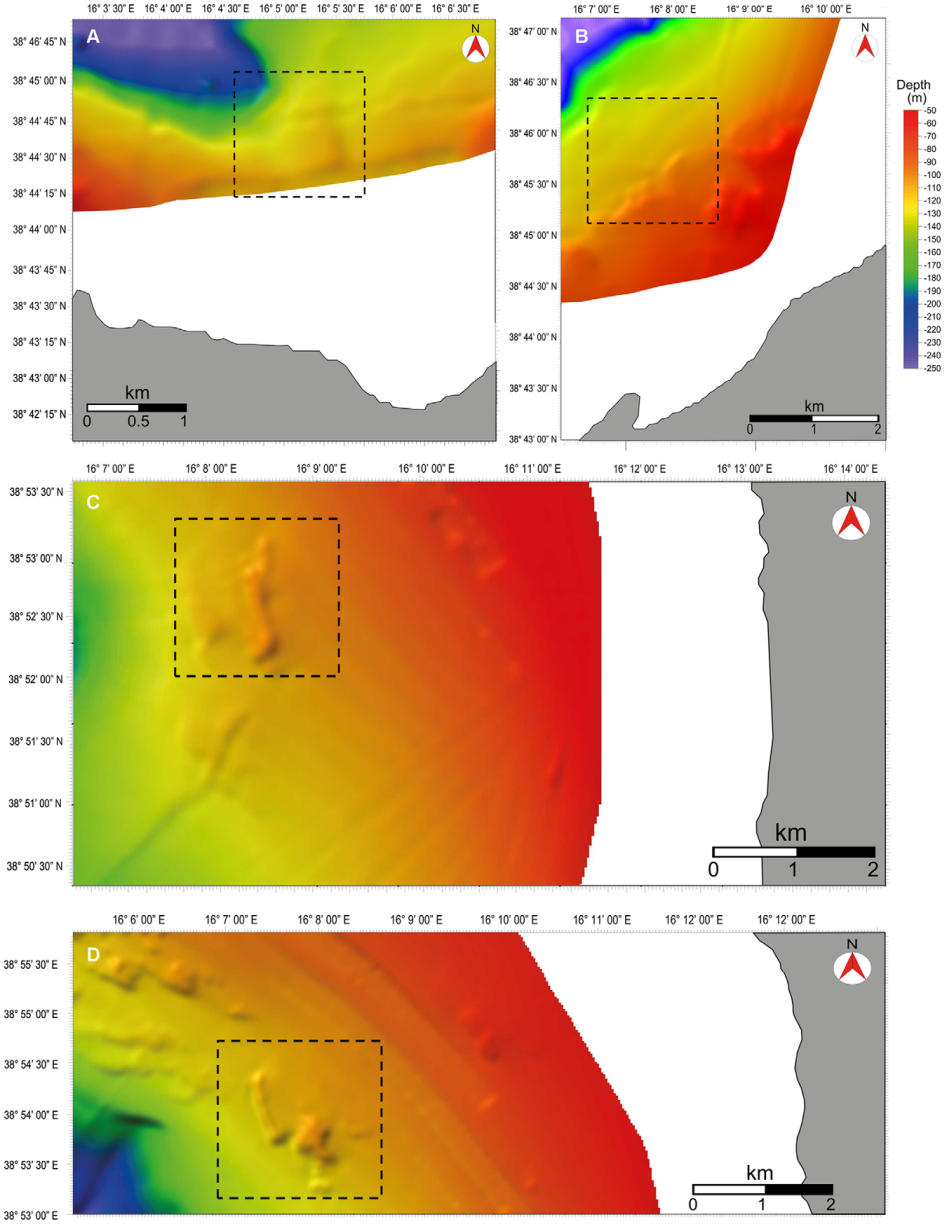
High resolution of morpho-bathymetric maps, including the studied shoals (dashed squares), obtained by using the Kingdom software (HIS Global Inc.). **A.** Shoal 1, **B.** Shoal 2, **C.** Shoal 3, **D.** Shoal 4.

Recent ROV investigations, mainly carried out along rocky vertical walls [8], [12], [19], [20], have disclosed the deep circa-littoral zone along the Calabrian continental shelf (southern Tyrrhenian Sea) as a hotspot of cold water coral biodiversity. The topography of this region, however, includes also wide gently sloping plains, such as the one found in the St. Eufemia gulf [25]. In particular, the sea floor of this area is spotted by numerous small rocky elevations emerging from a muddy bottom (Fig. 1) and representing the typical habitat of a “roche du large” biocoenosis.

**Figure 2:**
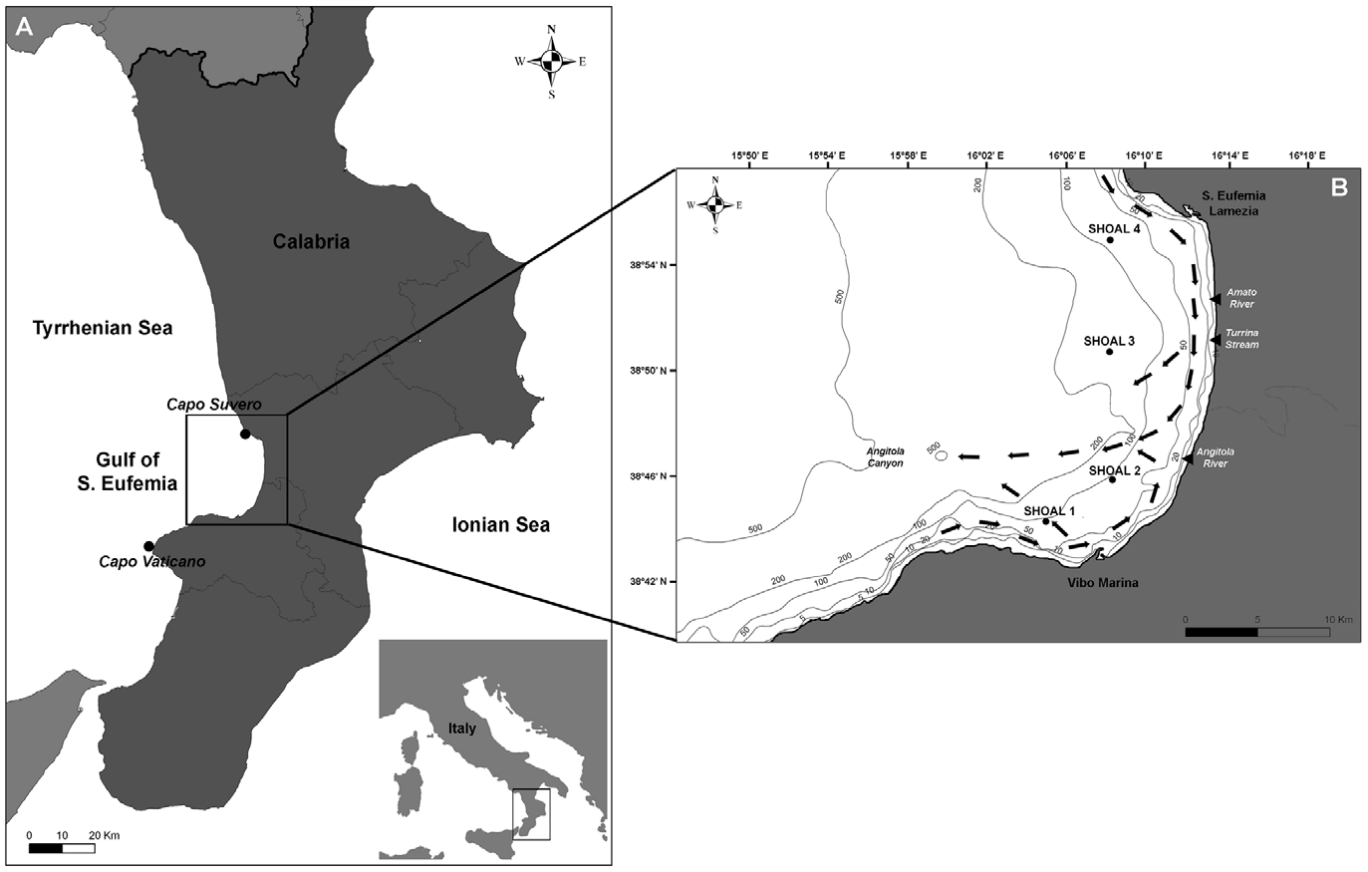
Location of St. Eufemia (A) and the shoals (B). The map shows also the location of the mouths of the principal rivers of the region and the circulation pattern (black arrows) of the coastal current directing towards the Angitola Canyon (modified from [26]).

The colonization of these rocky elevations and thus the composition of the associated benthic communities are likely controlled by a number of different co-occurring biotic and abiotic mechanisms. These mechanisms, in turn, may result in patterns of benthic biodiversity which could vary within small spatial scales, contributing to maintain high levels of diversity also at larger (basin) spatial scales. To infer about the ecological significance of “roche du large” communities, we explored their relevance as oases of biodiversity in the southern Tyrrhenian Sea. In particular, we tested the hypothesis that coral species abundance and community composition differ significantly among shoals located at a relatively narrow distance apart. In this study we used cnidarians as surrogate descriptors of megabenthic marine biodiversity due to their richness, abundance and identification easiness. We also explored the presence of signs of anthropogenic impacts to prompt the need of protection measures of these delicate deep oases of benthic biodiversity.

**Table 1:** Location of the shoals, number of ROV’s dives, photographed area and depth range of ROV’s explorations, distance of the shoals from the two main rivers entering the gulf and from the shore, number of pictures analyzed, corals counted and average abundance and species abundance per shoal.

	S1	S2	S3	S4	Total
**Coordinates**	38°44′32′′ N 16°05′22′′ E	38°45′33′′ N 16°08′14′′ E	38°52′15′′N 16°08′45′′E	38°54′07′′N 16°07′37′′E	
**Dives number**	15	8	5	6	34
**Photographed area (m^2^)**	343.5	276.0	186.1	183.8	989.4
**Depth range (m)**	70–110	95–130	70–120	70–120	70–130
**Distance from the Amato river (km)**	20	15.2	8.2	8.1	
**Distance from the Angitola river (km)**	10.5	5.3	9.4	16.8	
**Distance from the Turrina stream (km)**	17.2	12	7	10	
**Distance from the coast (km)**	2.4	3.8	7	3.8	
**Pictures number**	373	212	156	267	1008
**Counted corals**	3018	1085	308	1386	5797
**Abundance (n m^−2^± standard error)**	16.7±1.3	7.5±0.7	2.6±0.2	12.6±0.9	11.5±0.6
**Species abundance**	5	5	7	4	9

## Materials and Methods

### Ethics Statement

The ROV surveys and the sampling operations in the study areas of Calabria were conducted with regular permits (Aut. 79 ICRAM, R031250, Giu. 08– GE/DN7005; Aut. 96 ICRAM, R090737, Lug. 08; MARISTAT 2008 P261430Z; Reggio Calabria Port Authority N°15 2008) released by the Italian Navy Hydrographic Institute and by the Calabria Port Authorities.

**Table 2:** Results of the univariate analysis of variance testing for differences among the four shoals (S) in the abundance of each target coral and in the total coral abundance.

Species	Source	df	MS	F	P	Pairwise comparisons
Total coral	Shoal	3	62.60	75.14	***	S1>S4>S2>S3
	Residual	792	0.83			
*C. verticillata*	Shoal	3	23.87	153.19	***	S2>S3 Missing in S1 and S4
	Residual	792	0.16			
*E. cavolinii*	Shoal	3	63.45	101.21	***	S2>S4>(S1, S3)
	Residual	792	0.63			
*P. macrospina*-group	Shoal	3	162.62	108.92	***	S1>S4>S2>S3
	Residual	792	1.49			
*C. rubrum*	Shoal	3	0.26	4.27	**	S1>S2 Missing in S3 and S4
	Residual	792	0.06			
*A. dichotoma*	Shoal	3	0.70	16.29	***	S2>S3 Missing in S1 and S4
	Residual	792	0.04			
*P. clavata*	Shoal	3	24.11	45.73	***	S1>S4>S3 Missing in S2
	Residual	792	0.53			
*L. sarmentosa*	Shoal	3	0.14	6.27	**	S1>S4 Missing in S2 and S3
	Residual	792	0.02			
*P. larix*	Shoal	3	0.53	30.30	***	Exclusive of S1
	Residual	792	0.02			
*A. subpinnata*	Shoal	3	0.17	14.19	***	Exclusive of S3
	Residual	792	0.01			

df = degree of freedom; MS = mean square; P = probability level;

*** = P<0.001;

** = P<0.01. Reported are also the results of pairwise comparisons.

### Study Area

The gulf of St. Eufemia is a 42 km wide bay extending from North to South between Capo Suvero and Capo Vaticano (Fig. 2A) [26]. Two major rivers, Amato and Angitola, and the temporary stream Turrina, flow into the basin (Fig. 2B) [27]. The coastal current system is composed of various moderate intensity flows, moving in an anticyclonic gyre in the northernmost part of the gulf and in a cyclonic gyre in the southernmost region (Fig. 2B) [28]. Water masses, transporting the quartz-feldspar sands of fluvial origin, are mainly oriented through the Angitola Canyon, facing the river mouth, and flow deep over the Tyrrhenian bathyal plain [26] (Fig. 2B). The trophic characterization of the area indicates a general oligotrophic condition [25].

**Figure 3:**
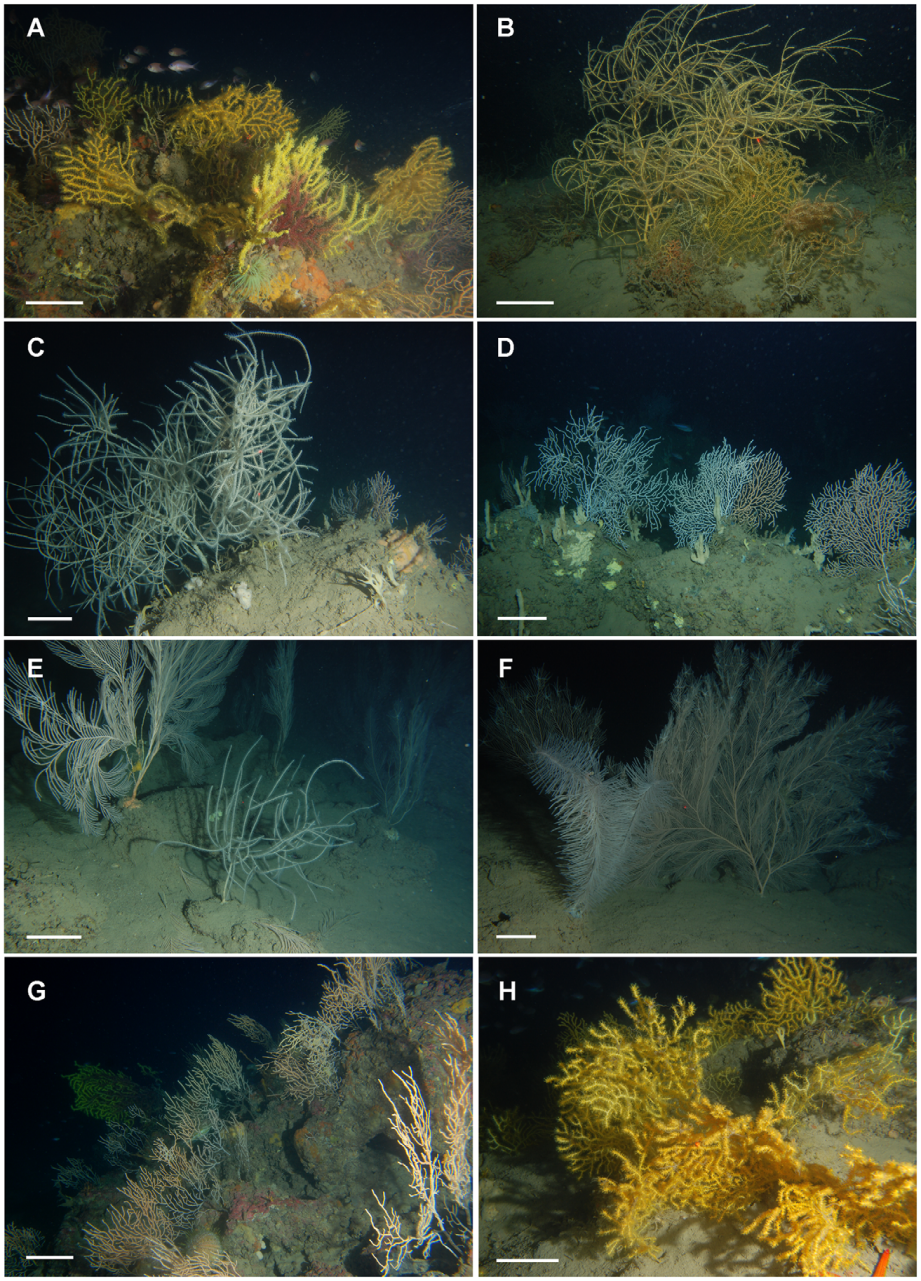
Coral assemblages of the studied shoals. Shoal 1 (A–B, 120 m depth): **A)** Mixed assemblage of *Paramuricea macrospina*, *Eunicella cavolinii* and *Paramuricea clavata*. **B)** Colonies of *P. macrospina* surrounding a colony of *Leptogorgia sarmentosa*. Shoal 2 –D, 120 m depth): **C)** Arborescent colony of *Antipathes dichotoma*, surrounded by small specimens of *E. cavolinii*. **D)** Fan-like specimens of *E. cavolinii* surrounded by tubular specimens of *Haliclona (Haliclona)* sp. Shoal 3 (E–F, 120 m depth): **E)** Fan-like specimens of *Callogorgia verticillata* and a juvenile colony of *A. dichotoma*. **F)** In the background, high colonies of *C. verticillata* spotted by numerous ophiuroids, while in the foreground a branched colony of the bottle-brush black coral *Parantipathes larix*. Shoal 4 (G–H, 120 m depth): **G)** Rocky elevation covered by *E. cavolinii* and *P. clavata*. **H)** Mixed assemblage of yellow colonies of *P. macrospina* and *P. clavata*. Scale bar: 10 cm.

**Figure 4:**
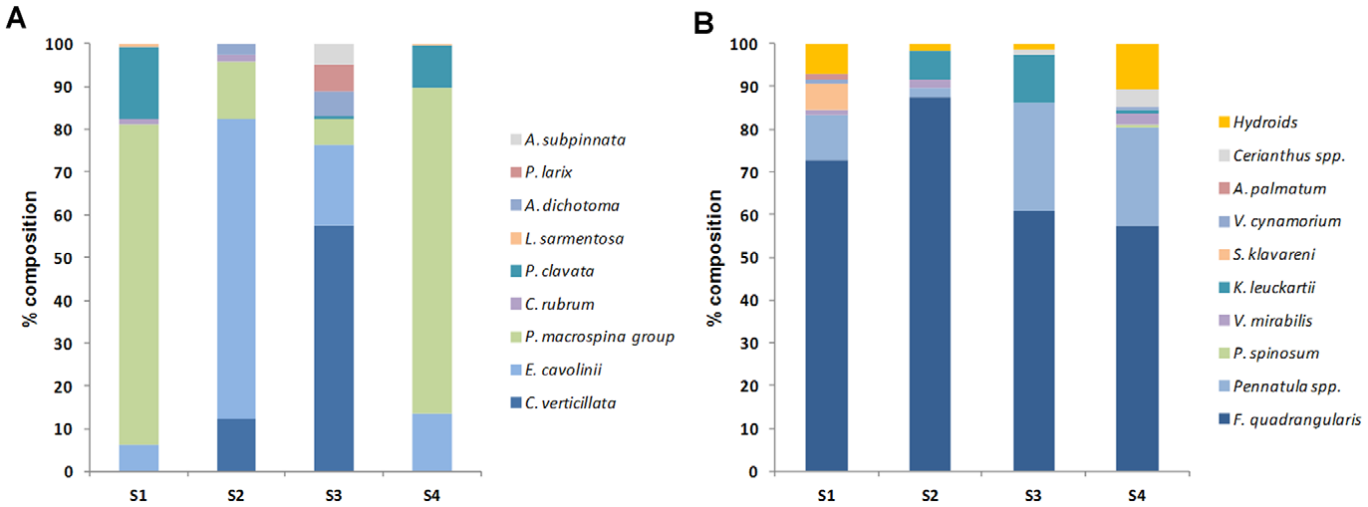
Percentage composition of the coral assemblages in A) the rocky shoals and B) their surrounding soft bottom.

The sea bottom is characterized by the presence of numerous small rocky shoals distributed over gently sloping muddy grounds as revealed by MultiBeam high resolution morpho-bathymetric data (Fig. 1A–D). We considered as a shoal an area composed of one or few major elevations (up to several m high) surrounded by mud and smaller, sparse rocks, at times partially covered by silt. Four shoals, namely S1, S2, S3 and S4 were identified within the study area (Figs. 1A–D, 2B), on average 5 km far from each other. Each shoal is characterized by a different set of features such as depth range, minimum distance from the coast, and distance from the main river and stream mouths (Amato, Angitola and Turrina) (Tab. 1). Shoal S2 was already partially described in previous studies [12], [29], which focused mainly on the description of a population of the black coral *Antipathes dichotoma* Pallas, 1766.

**Table 3:** Results of the PERMANOVA testing for the effects of shoal on the composition of the target hard-bottom corals assemblage.

PERMANOVA				
Source	df	MS	Pseudo-F	P
Shoal	3	271120	136.96	***
Residual	791	1980		
Total	794			
**Pairwise comparisons**				
**Contrast**		**t**	**P**	
S1 vs. S2		13.92	***	
S1 vs. S3		12.59	***	
S1 vs. S4		3.85	***	
S2 vs. S3		10,10	***	
S2 vs. S4		14.25	***	
S3 vs. S4		13.83	***	

*** = P<0.001. Reported are also the pairwise comparisons.

### Data Acquisition

Data were collected through a Remotely Operated Vehicle survey (ROV “Pollux”) conducted in June 2008, July 2008, and August 2009 on board of the R/V *Astrea* between 70 and 130 m depth. The ROV was equipped with a digital camera (Nikon D80, 10 megapixel), a strobe (Nikon SB 400), a high definition video camera (Sony HDR-HC7), and 3 jaw grabbers. The ROV hosted also a depth sensor, a compass, and two parallel laser beams providing a 10-cm scale for the measurement of the frames and the specimens’ size.

**Table 4:** Results of the SIMPER analyses showing dissimilarities between couples of shoals and the hard-bottom coral species mostly responsible for the observed differences.

Contrast	Dissimilarity (%)	Species	Contribution %	Cumulative %
S1 vs. S2	83.6	*P. macrospina*-g	36.6	36.6
		*E. cavolinii*	31.6	68.2
		*P. clavata*	16.9	85.1
		*C. verticillata*	9.1	94.2
S1 vs. S3	95.8	*P. macrospina*-g	39.0	39.0
		*C. verticillata*	20.2	59.2
		*P. clavata*	18.6	77.7
		*E. cavolinii*	12.8	90.5
S2 vs. S3	77.3	*E. cavolinii*	46.5	46.5
		*C. verticillata*	26.1	72.6
		*P. macrospina*-g	13.1	85.7
		*A. dichotoma*	5.8	91.6
S1 vs. S4	60.9	*P. macrospina*-g	50.5	50.5
		*P. clavata*	25.8	76.3
		*E. cavolinii*	20.1	96.4
S2 vs. S4	77.6	*P. macrospina*-g	43.6	43.6
		*E. cavolinii*	32.8	76.4
		*P. clavata*	9.9	86.3
		*C. verticillata*	9.7	96.0
S3 vs. S4	94.5	*P. macrospina*-g	46.7	46.7
		*C. verticillata*	20.1	66.8
		*E. cavolinii*	15.9	82.7
		*P. clavata*	10.1	92.8

The high resolution morpho-bathymetric maps (Fig. 1) were obtained by using a hull mounted MultiBeam echosounder (MBES), the Reson Seabat 8111 operating with 100 kHz of frequency and characterized by a nominal resolution of 3.7 cm. Data were acquired with 40% lateral overlap and processed to remove spikes due to navigation system problems and/or to the acquisition system. Acquisition and processing of data were performed using the PDS2000 packet. The final data were organized in a grid with cell size of 20×20 m.

**Table 5:** Results of the SIMPER analyses showing dissimilarities between couples of investigated areas and the soft bottom coral species mostly responsible for the observed differences.

Contrast	Dissimilarity (%)	Species	Contribution %	Cumulative %
S1 vs. S2	51.1	*K. leuckarti*	28.7	28.7
		*Pennatula* spp.	22.6	51.3
		*V. mirabilis*	13.4	64.7
		*F. quadrangularis*	12.7	77.4
		Hydroids	10.9	88.3
		*S. klavareni*	6.1	94.4
S1 vs. S3	49.9	*K. leuckarti*	27.9	27.9
		*Pennatula* spp.	24.6	52.5
		*F. quadrangularis*	12.7	65.2
		Hydroids	8.1	73.3
		*Cerianthus* spp.	7.7	81.1
		*V. mirabilis*	7.0	88.1
		*S. klavareni*	6.2	94.3
S2 vs. S3	42.1	*K. leuckarti*	30.9	30.9
		*Pennatula* spp.	22.3	53.1
		*F. quadrangularis*	14.5	67.6
		Hydroids	14.2	81.9
		*V. mirabilis*	9.6	91.4
S1 vs. S4	49.9	Hydroids	24.6	24.6
		*Pennatula* spp.	23.3	47.8
		*Cerianthus* spp.	12.4	60.2
		*V. mirabilis*	10.1	70.3
		*F. quadrangularis*	9.2	79.5
		*S. klavareni*	5.3	84.8
		*V. cynamorium*	5.0	89.8
		*P. spinosum*	4.2	94.1
S2 vs. S4	56.2	*Pennatula* spp.	26.3	26.3
		Hydroids	20.4	46.6
		*K. leuckarti*	19.1	65.7
		*Cerianthus* spp.	10.6	76.3
		*V. mirabilis*	9.5	85.8
		*F. quadrangularis*	8.0	93.8
S3 vs. S4	52.2	*Pennatula* spp.	23.2	23.2
		Hydroids	22.4	45.7
		*K. leuckarti*	20.0	65.6
		*Cerianthus* spp.	13.4	79.0
		*F. quadrangularis*	8.5	87.5
		*V. mirabilis*	5.9	93.4

The ROV was equipped with an underwater acoustic tracking position system (Tracklink 1500 MA, LinkQuest Inc.) providing detailed records of the tracks along the seabed. Tracks on each rocky shoal had an approximate total length of 9700 m (S1), 8400 m (S2), 7400 m (S3), and 8300 m (S4), covering a total explored area of about 50,000 m^2^ (the visual field of the ROV being 2 m when moving at a distance of 1.5 m from the bottom) (Tab. 1).

**Table 6:** Size of the hard bottom corals in the studied shoals.

Target corals	N° of counted colonies	Height × Width (cm)			
		S1	S2	S3	S4
*Eunicella cavolinii*	428	18±2×16±2	18±0.6×18±0.7	24±4×23±4	25±3.5×25±4
*Paramuricea clavata*	256	22±1×27±1		44±11×38±15	34±5×28±4.5
*Paramuricea macrospina*-group	1137	11±0.2×9.5±0.2	8±0.5×8±0.6	15±1×13±3	16±0.5×13±0.4
*Callogorgia verticillata*	145		40±3×31±3	45±3×46±4	
*Corallium rubrum*	50	6±0.6×4±0.6	9±2×9±1.5		
*Leptogorgia sarmentosa*	11	41±6.5×45±9.0			56±1×37±1
*Antipathella subpinnata*	10			21±5.5×28±7.5	
*Antipathes dichotoma*	35		62±6×81±5.5	57±5×72±9.5	
*Parantipathes larix*	19			41±6×20±3	

A total of 1008 high resolution photographs, taken on the rocky bottoms of the shoals during 34 dives, were analyzed (Tab. 1). Pictures were acquired every n seconds, with n = 60–180 sec (the value of n was extracted randomly before each dive). From each picture, the abundance (N° colonies m^−2^± SE) and community composition of coral taxa (see below) were estimated *a posteriori*. Pictures were used also for acquiring data about the colonies’ size (height × width, in cm) and to describe the population size structures of the most abundant species.

**Figure 5:**
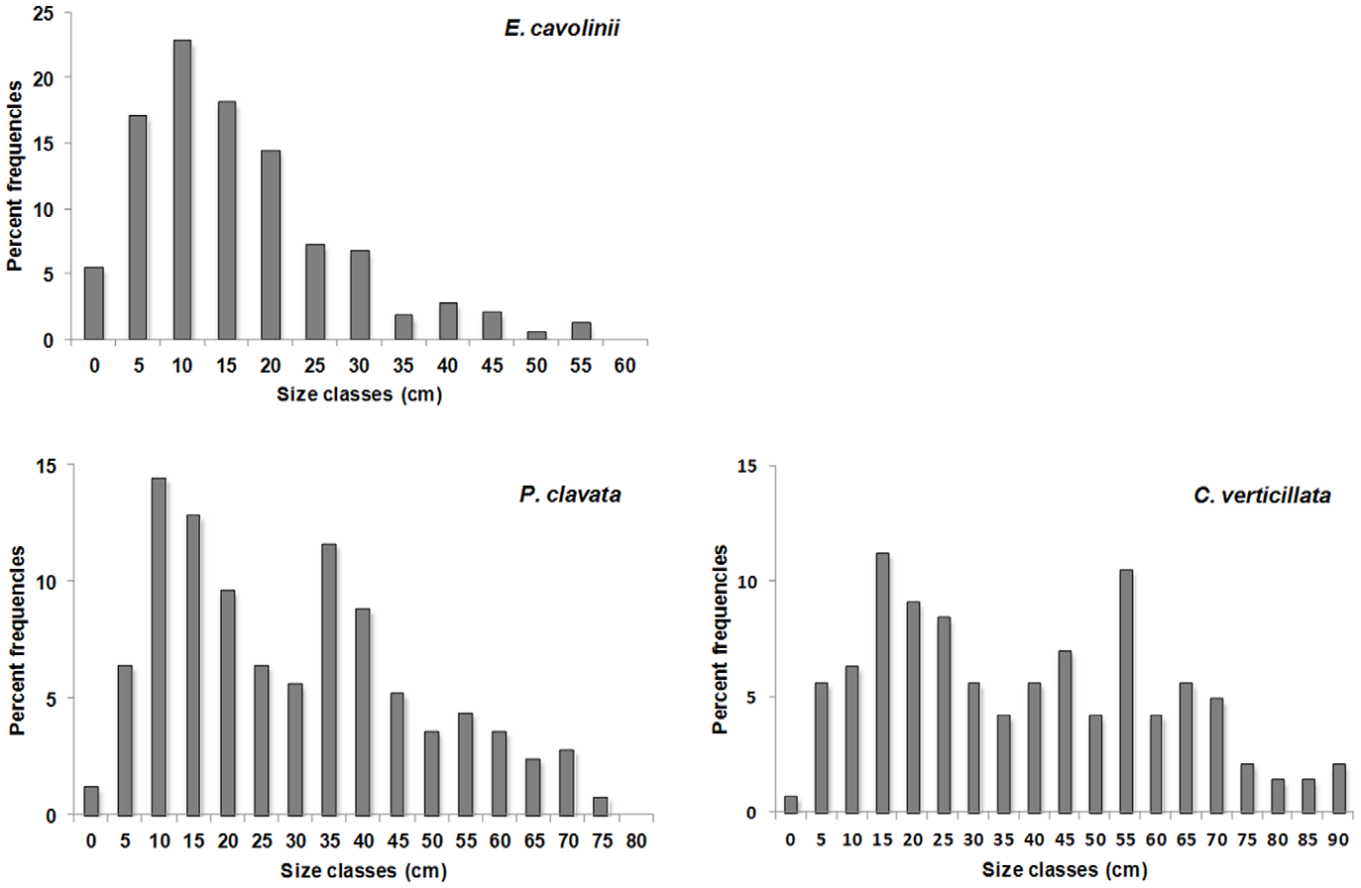
Size-frequency distribution of the most common hard bottom corals, namely *Eunicella cavolinii*, *Paramuricea clavata*, and *Callogorgia verticillata*.

**Figure 6:**
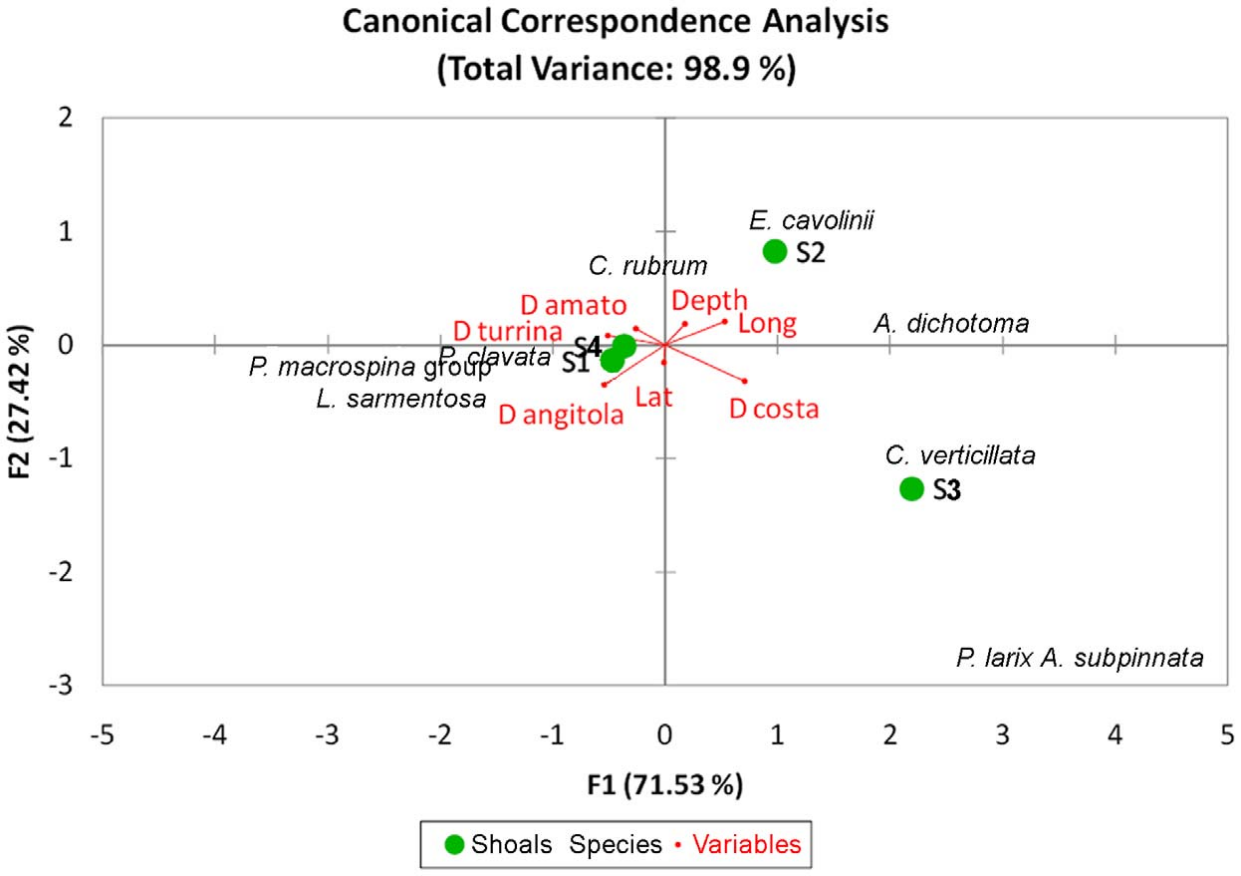
Results of the Canonical Correspondence Analysis.

**Figure 7:**
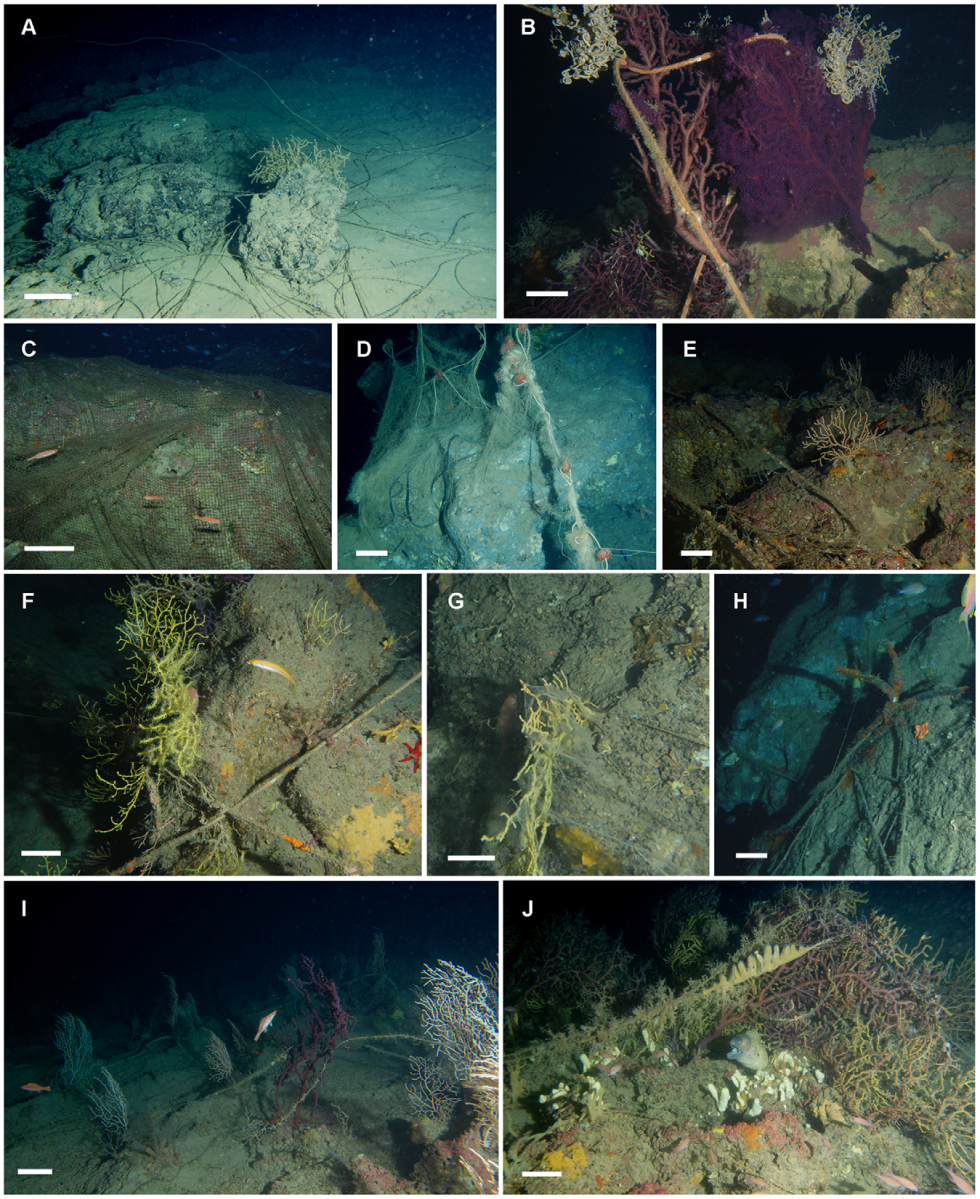
Impact of the fishing activity on the rocky shoals of St. Eufemia. A) Rocky boulders entrapped by lost long lines hosting colonies of *Bebryce mollis*. B) Colonies of *Paramuricea clavata* hosting the ophiuroids *Astrospartus mediterraneus* entangled by a rope peeling off the coenenchyme of the gorgonians. C–D) Ghost nets entirely wrapping rocky boulders. E) Lost net stretched over a population of *Eunicella cavolinii*. F–G) Abandoned long lines and plastic sheet entrapped in colonies of *P. clavata*. H) Abandoned anchor on the sea-floor. I) Lost lines stretching between a mixed assemblage of *E. cavolinii* and *P. clavata*. J) Old lines entangling colonies of *P. clavata* and covered by various epibionts such as sertulariid hydroids and ascidians. Scale bar: A, D: 20 cm, B–C, E–J: 10 cm.

To characterize the rocky shoals we considered the absolute and relative abundance of the most conspicuous coral species, corresponding to nine target taxa: the gorgonians *Callogorgia verticillata* (Pallas, 1766), *Corallium rubrum* (Linnaeus, 1758), *Eunicella cavolinii* (Koch, 1887), *Leptogorgia sarmentosa* (Linnaeus, 1758), and *Paramuricea clavata* (Risso, 1826), and the antipatharians *Antipathella subpinnata* (Ellis & Solander, 1786), *A. dichotoma* and *Parantipathes larix* (Esper, 1788). Specimens belonging to the gorgonians *Paramuricea macrospina* (Koch, 1882), *Bebryce mollis* Philippi, 1842 and *Villogorgia bebrycoides* (Koch, 1887) were pooled together and hereafter referred to the *P. macrospina-*group because they are scarcely distinguishable from each other on the basis of ROV images alone.

To characterize the coral fauna of the soft bottoms we analyzed also 8000 m^2^ of video transects recorded in the vicinity of the shoals. In each area, ten transects of 100 m length each (corresponding to about 200 m^2^ each) were carried out using the ROV to estimate the absolute and relative abundance of the soft bottom coral taxa. Ten taxa were considered, belonging to pennatulaceans (*Pteroides spinosum* (Ellis, 1764), *Funiculina quadrangularis* (Pallas, 1766), *Kophobelemnon leuckarti* Kölliker, 1872, *Pennatula* spp., *Virgularia mirabilis* (Müller, 1776)), gorgonians (*Spinimuricea klavareni* (Carpine & Grasshoff, 1975), alcyonaceans (*Veretillum cynomorium* Linnaeus, 1758, *Alcyonium palmatum* (Pallas, 1766)), ceriantharians (*Cerianthus* spp.) and hydroids (including, but not distinguishable from ROV images only, *Lytocarpia myriophyllum* (Linnaeus, 1758) and *Nemertesia antennina* (Linnaeus, 1758)).

All other identifiable benthic and ichthyic species found on and close to hard substrata were considered for each shoal assigning a relative abundance value when rarely (<10 observations per shoal), commonly (11–100 observations per shoal) or abundantly (>100 observations per shoal) observed in the examined photographic archive (Tab. S1).

The correspondence between the visual assignment of an individual to a certain coral species and its actual taxonomic classification was checked through the collection of at least one sample per species. Whole specimens, or portions of them, were used for morphological analyses and taxonomic classification. Samples were examined under light microscopy to study skeletal architectures and spicules/sclerites complements and, whenever necessary, portions or dissociates were mounted on stubs and sputtered with a 2 µm thick layer of gold-palladium. Micro-morphometric characteristics were observed under scanning electron microscopy (Phillips XL20).

To qualitatively characterize the presence of fishing gears in the investigated study areas, each analyzed picture was included in one of the following categories: A (no lost gear), B (presence of lost fishing lines and traps), and C (presence of lost nets), then a percentage was obtained for each group in each shoal. Moreover, the percentage of colonies, among the total counted ones in each shoal, that was evidently impacted by fishing gears was also annotated. To achieve a conservative measure of fishing impact, only the damaged colonies that were entangled by lines or nets were considered as correlated to a potential impact from fishing activities.

### Statistical Analyses

Prior to formal analyses, a Mantel’s test for spatial autocorrelation was carried out using the XLSTAT software. This test, exploring the correlation between the variations of coral community composition among the shoals and their geographical location, revealed that the community data were not affected by spatial autocorrelation (r = −0.550; P = 0.415). Thus, we proceeded to test the (null) hypothesis that abundance and composition of the coral communities do not differ among the four shoals, using either uni- or multi-variate analyses of variance. In both the uni- (abundance) and multivariate (composition) contexts, the design included the four different shoals (S1, S2, S3, S4) as the unique source of variation. Tests were carried out either on hard and soft-bottom communities separately using a distance-based permutational multivariate analysis of variance (PERMANOVA) with unrestricted permutation of data and permutation of residuals under the reduced model for the uni- and the multivariate analyses, respectively. The analyses were based on Bray-Curtis similarity of previously square root transformed data. Pairwise comparisons were also carried out wherever necessary to ascertain patterns in the abundance of the identified taxa or the assemblage composition among the four shoals. To identify the taxa better explaining the differences between the different shoals, SIMPER analyses were carried out on presence/absence transformed data using Bray-Curtis similarity matrices, separately for hard and soft-bottom communities.

Uni- and multivariate analysis of variance, PERMANOVA, and SIMPER analyses were carried out using routines included in the PRIMER6 + software package (Plymouth Marine Laboratory).

The relationships between hard bottom coral assemblages composition and some environmental factors were ascertained using a canonical correspondence analysis (CCA) carried out with XLstat software package. Explanatory variables comprised: latitude, longitude, distance from the coast, distance from the principal river mouths, and water depth.

## Results

Observations carried out by ROV allowed for the identification of rich hard and soft bottom megabenthic communities. Apart from the coral species, many other taxa were also recognized and recorded (Tab. S1, Figs. S1, S2). In particular, a total of 118 taxa were observed on the shoals and in their vicinity, with sponges (excluding encrusting taxa), cnidarians, mollusks and fish as the most abundant groups (from 10 to 30% of the total taxa) (Tab. S1). Of the four shoals, S1 and S4 hosted the higher number of identified taxa (69 and 64, respectively), followed by S2 and S3 (48 and 36, respectively).

### Hard Bottom Coral Fauna

The shoals in St. Eufemia hosted very rich coral assemblages whose abundance was, on average, 11.5±0.6 colonies m^−2^ calculated from a total of about 5,800 colonies counted through the image analysis (Tab. 1).

Total coral abundance varied significantly among the shoals (Tab. 2), with values in S1 and S4 (16.7±1.3 and 12.6±0.9 colonies m^−2^, respectively) being significantly higher than in the two other shoals (7.4±0.7 and 2.6±0.2 colonies m^−2^ for S2 and S3, respectively).

Shoal S1, the southernmost and closest to the coastline shoal, was dominated by the *P. macrospina-*group (13.6±1.3 colonies m^−2^) (Fig. 3A–B, Fig. 4A). Shoal S2, the closest to Angitola river’s outflow, hosted a mixed assemblage of gorgonians and antipatharians, with the dominance of *E. cavolinii* (4.9±0.5 colonies m^−2^) followed by the *P. macrospina-*group and *C. verticillata* (1.3±0.2 colonies m^−2^ and 0.9±0.2 colonies m^−2^, respectively), with only occasional colonies of the red coral *C. rubrum* and the black coral *A. dichotoma* (Fig. 3C–D, Fig. 4A). Shoal S3, the closest to Amato and Turrina’s outflows, hosted a complex assemblage of gorgonians and antipatharians, dominated by *C. verticillata* (1.4±0.2 colonies m^−2^) and *E. cavolinii* (0.6±0.1 colonies m^−2^). The other components (between 0.1±0.02 and 0.2±0.07 colonies m^−2^) were the *P. macrospina-*group, the bottle-brush black coral *P. larix*, *A. dichotoma* and *A. subpinnata*. The least represented taxon on this shoal was *P. clavata* (Fig. 3E–F, Fig. 4A). Finally, S4, the northernmost shoal, was characterized by a gorgonian assemblage dominated by the *P. macrospina-*group (9.5±0.9 colonies m^−2^). *E. cavolinii* and *P. clavata* (a characteristic totally yellow phenotype) were almost equally represented (1.6±0.1 and 1.4±0.2 colonies m^−2^, respectively), while *L. sarmentosa* was occasionally present (Fig. 3G–H, Fig. 4A).

Among the target corals, only *C. verticillata*, *E. cavolinii*, *P. clavata* and the *P. macrospina-*group displayed significant differences among shoals, though with variable patterns (Tab. 3). The abundance of all other corals was generally very low and did not varied significantly among shoals (Tab. 3). Considering only the target corals, S3 exhibited the highest coral species richness (7 species), followed by S1 and S2 (each one with 5 species), and, lastly, by S4 (4 species) (Tab. 1). The number of identified hard bottom taxa per m^−2^ varied among the four shoals between 1 and 4.

The *P. macrospina*-group (and within this, the species *P. macrospina*) was ubiquitous in all shoals, while the other species were encountered in only one or two shoals. In particular, *L. sarmentosa* was observed only in the most external shoals (S1 and S4), whereas *C. verticillata* and the black corals were observed in one or two of the intermediate shoals (S2 and S3).

The SIMPER analysis revealed that the differences in the hard-bottom coral fauna among the four shoals were mostly and consistently explained by variations in the abundance of the *P. macrospina*-group (Tab. 4).

### Soft Bottom Coral Fauna

The soft bottoms surrounding the rocky shoals were made up of mud with patches of organogenic detritus interspersed with fine sands and were mainly colonized by pennatulaceans (Fig. S2E–I). Occasionally, sponges (Fig. S2C), ceriantharians (Fig. S2D), gorgonians (Fig. S2J), soft corals (Fig. S2K–L), and large soft bottom hydroids (Fig. S2M–N) were also observed. Cidarids (Fig. S2P) and holothurians were also common, as well as various fish species (Fig. S2Q–V).

The total abundance of soft bottom corals was, on average, very low (0.06±0.01 colonies m^−2^ calculated from a total of about 461 specimens counted through the whole video analysis). PERMANOVA revealed that, among the four soft bottom areas, there are significant differences in coral abundance and richness (Tab. S2). *F. quadrangularis* was the most abundant species (up to 15.4±3.17 colonies m^−2^ in S2), followed by *Pennatula* spp. (up to 2.8±0.74 colonies m^−2^ in S4), hydroids and *K. leuckarti* (up to 1.3±0.34 colonies m^−2^ in S4 and up to 1.2±0.51 colonies m^−2^ in S2, respectively) (Fig. 4B).

The average abundance of soft bottom taxa (0.01±0.01 taxa m^−2^) was up to three orders of magnitude smaller than that in hard bottoms (2.65±0.13 taxa m^−2^).

The SIMPER test revealed that the dissimilarity in the composition of soft bottom corals among the four studied areas (ranging 42–56%; Tab. 5) is considerably lower than that observed in terms of hard-bottom corals (range 77–96%; Tab. 4).

### Population Structure of Hard Bottom Corals

The average size of each hard bottom target coral species, obtained from the measures of 2091 coral colonies, are summarized in Table 6.

Height measures were used to obtain the size-frequency distributions describing the population structure of the most abundant coral species (Fig. 5). The size-frequency distribution of *E. cavolinii* is unimodal with a mode in the 10–15 cm size class and a long tail of rare large colonies (max 57 cm high) (Fig. 5).

A different situation is observed for *P. clavata* and *C. verticillata*. Both these species have a multimodal size-frequency distribution: the two main modes of *P. clavata* are in the ranges 10–15 cm and 35–40 cm, respectively (Fig. 5) while those of *C. verticillata* are in the ranges 15–20 cm and 55–60 cm, respectively. Both species show a long tail of rare large specimens (max 80 and 95 cm high, respectively) (Fig. 5).

Species size structures were similar when considering separately the different shoals (data not shown).

### Factors Controlling Hard Bottom Corals

The Canonical Correspondence Analysis (CCA) revealed that the combination of all included environmental factors explains about 99% of the total variations in the composition of target coral assemblages of the four shoals. The bi-plot reveals an evident segregation between S1 and S4, characterized by a greater abundance of the *P. macrospina*-group, *P. clavata* and *E. cavolinii* and S2 and S3, both characterized by the exclusive presence of *C. verticillata* and black coral species (Fig. 6). All tested explanatory variables contributed significatively to the discrimination of the four shoals in terms of assemblage composition.

Among the other factors that may have an influence on the coral assemblages of the shoals we also took into consideration, though from a mere qualitative point of view, the fishing impact. The rocky shoals in St. Eufemia, in fact, host numerous commercially relevant crustacean (Fig. S2O) and fish (Fig. S2Q) species, that, due also to the off-coast localization of these habitats, attract the local recreational and professional fishing boats. On the basis of the image analysis, the highest percentage of pictures (between 88–96%) fell in the category A corresponding to no visible lost gears. Gears falling in the category B, lines and traps, were observed in 4–10% of the analyzed pictures, whereas only up to 3% of the images were assigned to category C, corresponding to lost nets (Fig. 7). Only 1.3% of the counted specimens were evidently damaged by fishing gears.

## Discussion and Conclusions

Dense assemblages of benthic cnidarians have been studied above 50 m depth in numerous regions of the western Mediterranean Sea [23], [30], [31], [32], [33], but only few studies have been carried out deep towards the limits of the continental shelf [4], [7], [8]. In this regard, the observations conducted in the gulf of St. Eufemia greatly enhance the knowledge on the structure and composition of the “roche du large” biocoenoses. On the basis of numerous recent ROV and diving surveys, these ecosystems are increasingly being recorded, even emerging as widely distributed habitats along the entire rocky circa-littoral depth range of the whole Mediterranean Sea [34]. This in the case for black corals, which have for a long time been considered to be among the least-known Mediterranean coral species, but occur here in discrete populations, hence raising important conservation concerns [12], [35].

The rocky habitats of St. Eufemia show a heterogeneous distribution, where the hard bottoms are represented by small and sparse boulders emerging from an almost flat muddy bottom characterized by a slightly turbulent environment. All the explored shoals are covered by silt and this might explain the low abundance of encrusting taxa, such as sponges, bryozoans and ascidians that are well represented only on dead coral branches or on the largest boulders with slightly steeper flanks. The dominant corals in St. Eufemia are those belonging to the *P. macrospina*-group; these gorgonians apparently behave as opportunistic species, able to colonize various types of either natural or artificial substrates. In the study areas, it is indeed not rare to observe colonies of these species growing on dead portions of other corals or on abandoned wastes on muddy bottoms (Fig. S1I, T). This suggests that, probably in relation to their relatively fast growth rates, they have a larger adaptability than other gorgonians and antipatharians. This, in turn, might explain their large dominance throughout the investigated area.

Some species are known to be well adapted to this environment, *L. sarmentosa*, in particular, is a suspension feeder typical of the detritic benthic Mediterranean communities colonizing habitats subjected to slow currents [36], [37], [38], [39]. In such slightly turbulent conditions, some coral species may reach a large size, as observed for *C. verticillata* (fan over 1.5 m tall), *A. dichotoma* (colonies over 1.5 m wide) and *P. larix* (colony over 1.2 m tall). On the other hand, the colonies of *A. subpinnata*, which grow more than 1 m in height along vertical walls lashed by strong currents [8], do not exceed 30 cm in St. Eufemia and are less pinnulated [29] probably as an adaptation to moderate currents and high silting.

The three considered species show a similar size structure among the shoals in which they are present, suggesting that their population structure is stable on the different shoals. In terms of species, the size-frequency distributions indicate a difference between the population structure of the small-sized species *E. cavolinii* and that of the large-sized ones (*P. clavata* and *C. verticillata*). The former is dominated by small colonies with few large individuals indicating a high adult mortality and/or the possibility to reach large sizes only in peculiar micro-habitats [40]. This kind of population structures was recorded also in the shallow-water populations of *P. clavata* and *Eunicella singularis* (Esper, 1791) studied along the Spanish coast of the Mediterranean Sea [23], [41]. On the contrary, the population structures of *P. clavata* and *C. verticillata* of St. Eufemia show some distinct cohorts of colonies with gaps of intermediate-sized specimens. This situation can be attributed to a discontinuous recruitment occurring in deep habitats. Similar differences between species living in deep habitats were recorded for *Primnoa resedaeformis* (Gunnerus, 1763) and *Paragorgia arborea* (Linnaeus, 1758) showing an unimodal and a multimodal size distributions, respectively [42], [43].

One of the major distinctive features of St. Eufemia deep circa-littoral coral communities is the coexistence, within a 25 km radius, of rocky elevations characterized by significantly different coral communities. In this sense, the shoals represent ecological analogues of islands as described in the theory of island biogeography [44]. According to this theory, the species richness of natural communities living in suitable habitats surrounded by unlike ecosystems is influenced by several main factors: the flux of organisms and larvae (depending on the distance from sources and hence the degree of their isolation), the size of the island (influencing the habitat heterogeneity and competition outcomes), the oceanographic supplies, serendipity (chance arrivals) and human activities [44]. Most likely, the extension of soft sediments in the deep sea creates a major dispersal barrier for hard bottom benthic organisms, thus favoring the existence of isolated habitats [45], [46], mainly in the form of discrete primary (natural or anthropogenic) or secondary hard substrata [47], [48].

The distribution of coral species on the shoals of St. Eufemia recalls many of the above-mentioned aspects: very dense aggregations of species living in very small hard grounds and surrounded by otherwise homogeneous soft bottoms with a greatly dispersed coral assemblage. The comparison between the shoals and the surrounding soft bottoms indicates differences from four to three orders of magnitude for coral abundance and β-diversity respectively, strongly supporting the identification of the rocky shoals of St. Eufemia as oases of biodiversity.

CCA reveals that the considered environmental variables almost equally explain the differences in terms of coral composition among the four investigated shoals. Thus it is possible to hypothesize that the distance from the river mouths, hence silting and trophic loads, may have a major contribution. There are several additional factors, apart from the tested ones, that could contribute to create and maintain large differences in the coral abundance and composition among the shoals in St. Eufemia. Many Mediterranean colonial cnidarians, for example, rely on creeping or short-distance swimming larvae, resulting in a limited larval dispersal ability [32], [49], [50], [51], [52], [53], [54], [55]. However, the alternate anti-cyclonic and cyclonic gyres existing in the gulf [26] most probably cause highly variable oceanographic conditions which, in turn, cause the occurrence of variable larval transport and settling patterns. Finally, settling success in a patchy rocky habitat depends also on the competition and adaptation abilities of each species once settled. For example, some of the coral species in St. Eufemia shoals, such as *E. cavolinii*, *P. clavata* and *A. subpinnata*, which on steep vertical walls elsewhere are generally distributed in distinct depth ranges [8], probably do not have enough space here to differentiate their vertical niches. This would explain the co-existence of otherwise distributed species within the same depth interval of the heterogeneous hard ground of the shoals in St. Eufemia,.

Fishing activities may have profound effects on hard bottom communities, either directly damaging the coral colonies or indirectly influencing their associated fauna [23], [56], [57], [58], [59], [60]. Our study reveals that the coral assemblages of St. Eufemia’s shoals are subjected to a relatively limited impact from bottom fisheries. Some of the damaged erect filter feeders show signs of wrecking or, as in the case of several specimens of *C. verticillata*, were deeply abraded and covered by epibionts. Various factors may increase the susceptibility of a certain species to the damage; for example, its size may increase the chance to remain entangled (as in the case of the species displaying tall colonies, such as *P. clavata* and *C. verticillata*) or its flexibility may increase the resistance to mechanical friction.

Altogether, the presence of large and significant differences in the coral assemblages composition (i.e. β-diversity) among the four shoals, the occurrence of several rare coral species, the susceptibility of the coral communities to critical environmental conditions and the existence of a certain – though not yet compromising - fishing pressure, underline that the shoals constellating the soft muddy sediments contribute to maintain high levels of γ-diversity in the St. Eufemia gulf. As such they can be considered highly vulnerable habitats and deserve special attention in a protection perspective. Our results, in fact, point out that “roche du large” biocoenoses can have an extraordinary role in controlling benthic biodiversity of the circa-littoral Mediterranean Sea at levels and spatial scales comparable to those identified for deep water corals, their surroundings and even for coral rubble patches [61]. Nevertheless, while deep water living coral reefs (but not coral rubble patches; [61]) are now included within the EUNIS habitat classification for the deep sea (http://www.eunis.eea.europa.eu/01/06/) and the UN Resolution attempting to protect vulnerable marine ecosystems (VMEs) from destructive fishing practices in international waters (A/RES/61/105), no special regional, national or international legislation or resolution manifestly asks for the protection of “roche du large” coral assemblages in the deep circa-littoral zone.

## Supporting Information

Figure S1 **Hard bottom fauna.** Shoal 1 (A–E): A) Various phenotypes of *Paramuricea macrospina*. Echinoderms found in the proximity of the coral colonies. B) Bryozoan *Turbicellepora avicularis* (Hincks, 1860) attached to *P. macrospina*. C) Rocky bottom hosting erect specimens of the sponge *Axinella* spp. and *Corallium rubrum*. D) Branched specimen of the pink sponge *Haliclona* (*Soestella*) *implexa* (Schmidt, 1868) on a rock covered by red algae. E) Sandy patch hosting ceriantharians and burrowing ophiuroids. Shoal 2 (F–J): F) Colonized dead colony of *Callogorgia verticillata*. Are visible numerous hydroids and alcyonaceans covering the branches. G) *Haliclona* (*Haliclona*) sp. forming branched tubular structures. H) Epibionts living on both living and dead portions of *C. verticillata*, such as crinoids, hydroids, solenogastres, ovulids and nudibranchs. I) Patchy distribution of corals on S2, with mixed sponge and coral assemblage. J) Yellow branches of the sponge *Suberites syringella*. Shoal 3 (K–O) K) Branched colony of *Parantipathes larix*, creating refuge for the fish *Lappanella fasciata* (L). M) Ophiuroids hanging on the ramifications of *C. verticillata*. N-O) Mix assemblage of gorgonians and antipatharians on an heavily sedimented rocky floor. Shoal 4 (P–T): P) School of *Anthias anthias* (Linnaeus, 1758) and *Callanthias ruber* (Rafinesque, 1810) swimming over the gorgonian assemblage. Q) Rocky bottom hosting red algae, massive and encrusting sponges and coral colonies. R) Specimens of the blue alcyonacean *Paralcyonium spinulosum* Delle Chiaje, 1822. S) *P. macrospina* hosting an unidentified ovulid (close-up in the inset). T) *P. macrospina* colonies growing on an abandoned tyre in a muddy patch. Scale Bar: A–C, E–K, M–R, T: 10 cm; D, L: 5 cm; S: 2.5 cm; S inset: 0.5 cm.
(TIF)

Figure S2 **Soft bottom fauna.** Sponges (A–C): A) Branched specimen of *Raspailia viminalis* Schmidt, 1862. B) Tubular specimen of *Calyx nicaeensis* (Risso, 1826). C) Group of stalked *Rhizaxinella pyrifera* (Delle Chiaje, 1828) on soft bottom. Cnidarians (D–N): D) *Cerianthus* sp. Pennatulaceans (E–I): E) *Pteroides spinosum*, F) *Kophobelemnon leuckartii*, G) *Pennatula rubra* (Ellis, 1764), H) *Funiculina quadrangularis*, I) *Virgularia mirabilis*. J) Branched specimen of the gorgonian *Spinimuricea klavareni*. K) *Alcyonium palmatum*. L) *Veretillum cynomorium*. Colonies of the soft bottom hydroids M) *Nemertesia antennina* and N) *Lytocarpia myriophyllum*. Crustaceans: O) *Palinurus elephas* (Fabricius, 1787) moving in a gorgonian meadow. Echinoderms: P) Assemblage of cidarid echinoids. Fish (Q–V): Q) *Zeus faber* (Linnaeus, 1758), R) school of *Macroramphosus scolopax* (Linnaeus, 1758), S) population of *Cepola macrophthalma* (Linnaeus, 1758) and detail of a specimen in the inset, T) specimens of *Helicolenus dactylopterus* (Delaroche, 1809), U) specimen of *Phycis phycis* (Linnaeus, 1766), V) shark *Scyliorhinus stellaris* (Linnaeus, 1758). Scale bar: I, S inset: 2.5 cm; B, K, L: 5 cm; A, C– G, J, M–R, T–U: 10 cm; H, S, V: 20 cm.
(TIF)

Table S1 **List and relative abundance of the species identified in the gulf of St. Eufemia.**
(DOC)

Table S2 **Results of the PERMANOVA testing for the effects of area on the abundance, taxa richness and composition of the target soft bottom corals.**
(DOC)
