# Supplementary figures and images for: Deep Coral Oases in the South Tyrrhenian Sea
Source: PLoS One. 2012 Nov 21;7(11):e49870. doi: 10.1371/journal.pone.0049870 (PMC3503811; doi:10.1371/journal.pone.0049870)

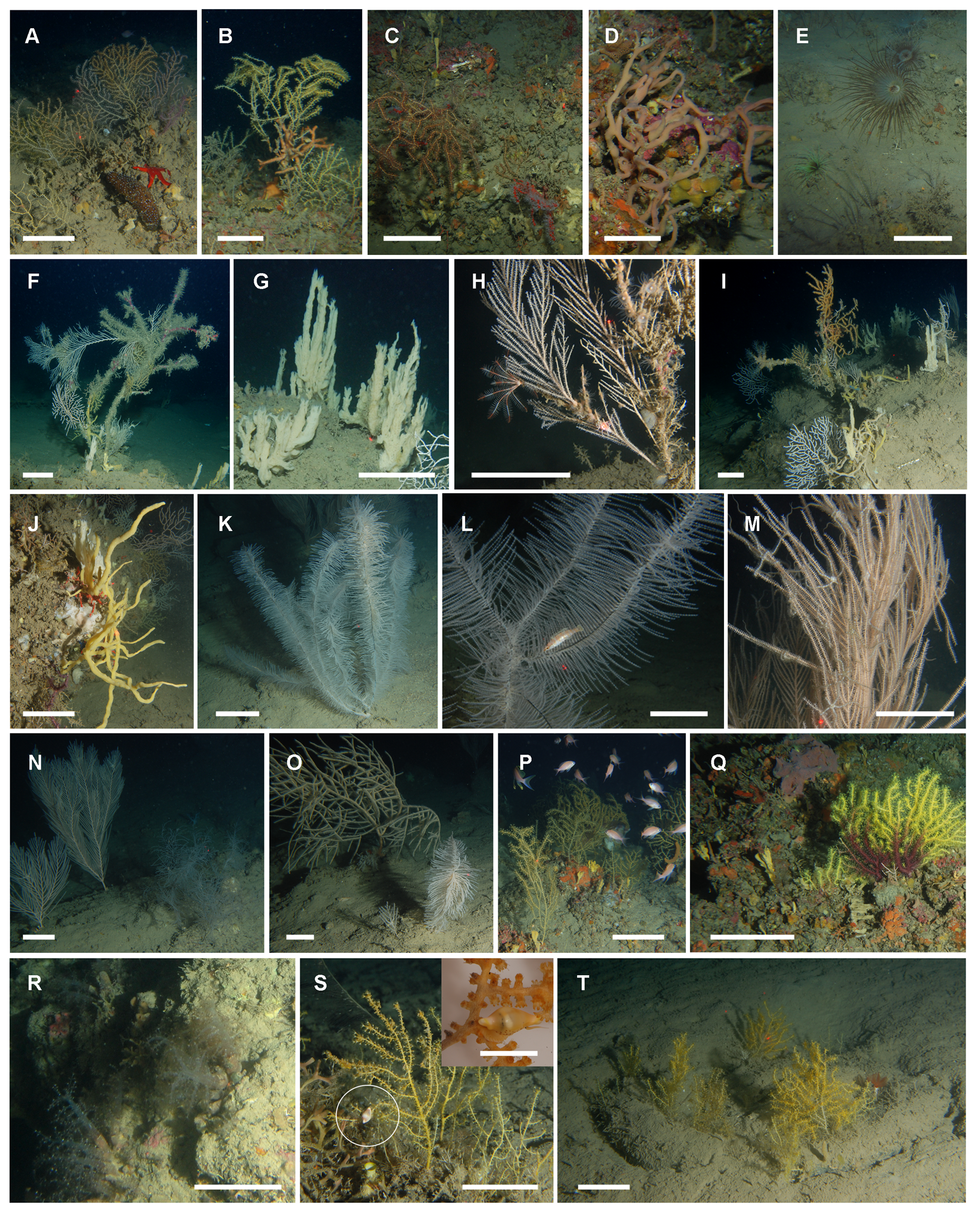

Supplement: Figure S1 — Hard bottom fauna. Shoal 1 (A–E): A) Various phenotypes of Paramuricea macrospina. Echinoderms found in the proximity of the coral colonies. B) Bryozoan Turbicellepora avicularis (Hincks, 1860) attached to P. macrospina. C) Rocky bottom hosting erect specimens of the sponge Axinella spp. and Corallium rubrum. D) Branched specimen of the pink sponge Haliclona (Soestella) implexa (Schmidt, 1868) on a rock covered by red algae. E) Sandy patch hosting ceriantharians and burrowing ophiuroids. Shoal 2 (F–J): F) Colonized dead colony of Callogorgia verticillata. Are visible numerous hydroids and alcyonaceans covering the branches. G) Haliclona (Haliclona) sp. forming branched tubular structures. H) Epibionts living on both living and dead portions of C. verticillata, such as crinoids, hydroids, solenogastres, ovulids and nudibranchs. I) Patchy distribution of corals on S2, with mixed sponge and coral assemblage. J) Yellow branches of the sponge Suberites syringella. Shoal 3 (K–O) K) Branched colony of Parantipathes larix, creating refuge for the fish Lappanella fasciata (L). M) Ophiuroids hanging on the ramifications of C. verticillata. N-O) Mix assemblage of gorgonians and antipatharians on an heavily sedimented rocky floor. Shoal 4 (P–T): P) School of Anthias anthias (Linnaeus, 1758) and Callanthias ruber (Rafinesque, 1810) swimming over the gorgonian assemblage. Q) Rocky bottom hosting red algae, massive and encrusting sponges and coral colonies. R) Specimens of the blue alcyonacean Paralcyonium spinulosum Delle Chiaje, 1822. S) P. macrospina hosting an unidentified ovulid (close-up in the inset). T) P. macrospina colonies growing on an abandoned tyre in a muddy patch. Scale Bar: A–C, E–K, M–R, T: 10 cm; D, L: 5 cm; S: 2.5 cm; S inset: 0.5 cm. (TIF) [file pone.0049870.s001.tif]

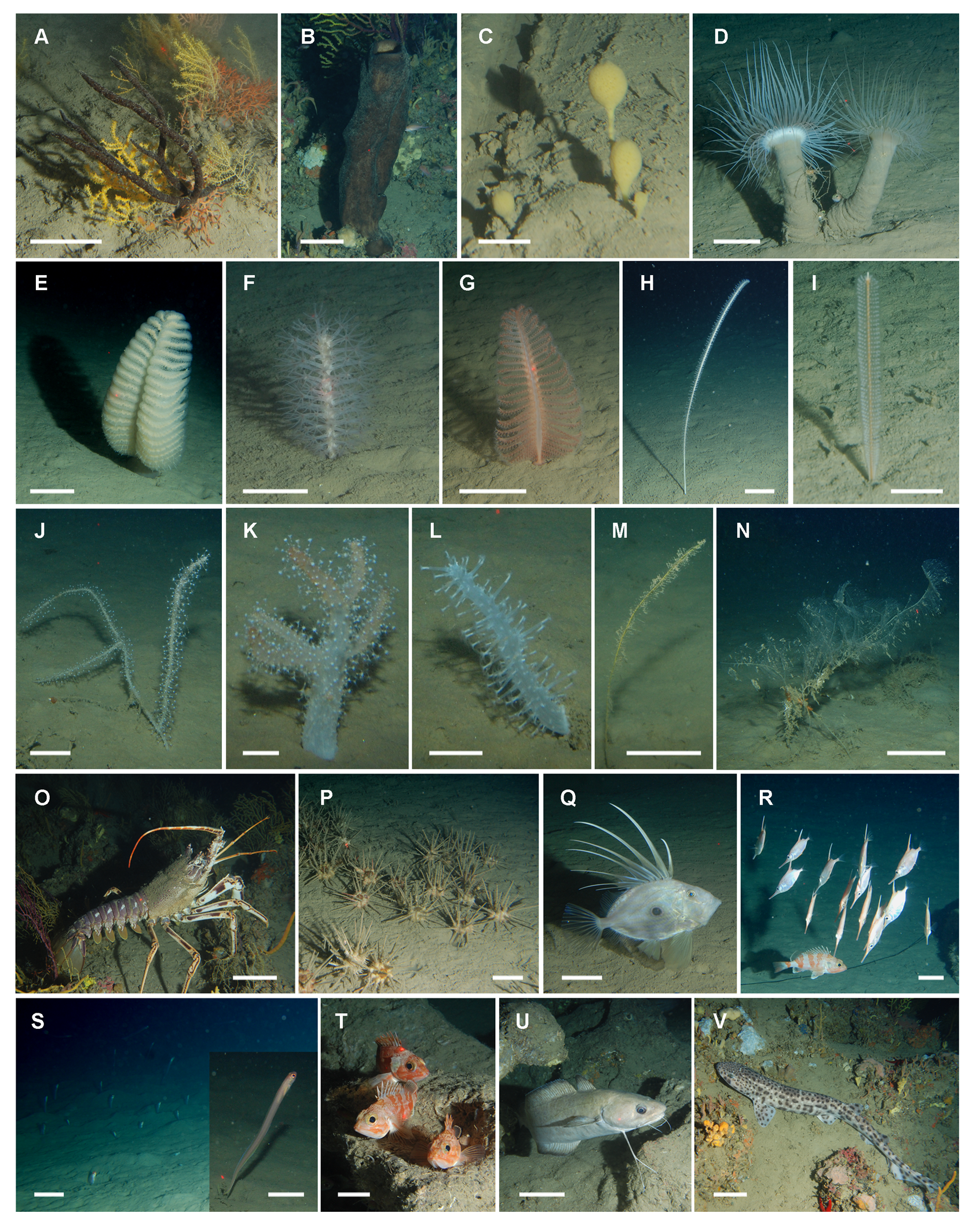

Supplement: Figure S2 — Soft bottom fauna. Sponges (A–C): A) Branched specimen of Raspailia viminalis Schmidt, 1862. B) Tubular specimen of Calyx nicaeensis (Risso, 1826). C) Group of stalked Rhizaxinella pyrifera (Delle Chiaje, 1828) on soft bottom. Cnidarians (D–N): D) Cerianthus sp. Pennatulaceans (E–I): E) Pteroides spinosum, F) Kophobelemnon leuckartii, G) Pennatula rubra (Ellis, 1764), H) Funiculina quadrangularis, I) Virgularia mirabilis. J) Branched specimen of the gorgonian Spinimuricea klavareni. K) Alcyonium palmatum. L) Veretillum cynomorium. Colonies of the soft bottom hydroids M) Nemertesia antennina and N) Lytocarpia myriophyllum. Crustaceans: O) Palinurus elephas (Fabricius, 1787) moving in a gorgonian meadow. Echinoderms: P) Assemblage of cidarid echinoids. Fish (Q–V): Q) Zeus faber (Linnaeus, 1758), R) school of Macroramphosus scolopax (Linnaeus, 1758), S) population of Cepola macrophthalma (Linnaeus, 1758) and detail of a specimen in the inset, T) specimens of Helicolenus dactylopterus (Delaroche, 1809), U) specimen of Phycis phycis (Linnaeus, 1766), V) shark Scyliorhinus stellaris (Linnaeus, 1758). Scale bar: I, S inset: 2.5 cm; B, K, L: 5 cm; A, C– G, J, M–R, T–U: 10 cm; H, S, V: 20 cm. (TIF) [file pone.0049870.s002.tif]
