# Supplementary material for: Deep Coral Oases in the South Tyrrhenian Sea
Source: PLoS One. 2012 Nov 21;7(11):e49870. doi: 10.1371/journal.pone.0049870 (PMC3503811; doi:10.1371/journal.pone.0049870)
Supplement: Table S1 — List and relative abundance of the species identified in the gulf of St. Eufemia. (DOC) [file pone.0049870.s003.doc]

**Table S1.** List and relative abundance of the species identified in the gulf of St. Eufemia.

| **Taxa (118)** | **Relative abundance** | | | |
| --- | --- | --- | --- | --- |
|  | **S1** | **S2** | **S3** | **S4** |
| **ALGAE (1)** |  |  |  |  |
| Rhodoficea | * |  |  | * |
| **PORIFERA (13)** |  |  |  |  |
| *Aplysina cavernicola* | * |  |  | * |
| *Axinella* spp. | *** | ** | * | *** |
| *Calyx nicaeensis* |  |  |  | * |
| *Clathrina clathrus* | * |  |  |  |
| *Chondrosia reniformis* | * |  |  |  |
| *Dysidea* spp. | * |  |  |  |
| *Haliclona (Haliclona) magna* |  | ** | * |  |
| *Haliclona (Haliclona)* sp. | ** | *** | * | * |
| *Haliclona (Soestella) implexa* | ** |  |  | ** |
| *Hexadella racovitzai* |  |  |  | * |
| *Raspailia viminalis* |  |  |  | * |
| *Rhizoaxinella pyrifera* |  | * |  |  |
| *Suberites syringella* |  | * |  | * |
| **CNIDARIA (38)** |  |  |  |  |
| Aglaophenidae g.sp. |  |  |  | * |
| *Alcyonium acaule* |  |  |  | * |
| *Alcyonium coralloides* |  | * |  | * |
| *Alcyonium* cf. *elegans* | * |  |  | * |
| *Alcyonium palmatum* | * | * | * |  |
| *Alicia mirabilis* | * |  |  |  |
| *Amphianthus* sp. |  | ** | ** |  |
| *Antennella* sp. |  | * |  |  |
| *Antipathella subpinnata* |  |  | ** |  |
| *Antipathes dichotoma* |  | * | ** |  |
| *Aurelia aurita* | * |  |  | * |
| *Bebryce mollis* |  | * |  |  |
| Bougainvillidae g. sp. |  | * | * |  |
| *Callogorgia verticillata* |  | ** | *** |  |
| *Cerianthus* spp. | ** |  |  |  |
| *Corallium rubrum* | * | * |  |  |
| *Dendrophyllia cornigera* | * | * |  |  |
| *Ectopleura* sp. |  |  | * |  |
| *Epizoanthus* sp. | * | * | * | * |
| *Eunicella cavolinii* | * | *** | ** | * |
| *Funiculina quadrangularis* |  | * | * | * |
| *Kophobelemnon leukarti* |  | * | * | * |
| *Leptogorgia sarmentosa* | * |  |  | * |
| *Lytocarpia myriophyllum* | * |  | * | * |
| *Nemertesia antennina* | * |  |  | * |
| *Obelia longissima* |  |  |  |  |
| *Paralcyonium spinulosum* | * | ** | * | * |
| *Parantipathes larix* |  |  | * |  |
| *Paramuricea clavata* | * |  | * | * |
| *Paramuricea macrospina* | *** | * | * | * |
| *Parazoanthus axinellae* | * |  |  | * |
| *Pennatula phosphorea* |  |  | * | * |
| *Pennatula rubra* | * |  |  | * |
| *Pteroides spinosum* |  |  |  | * |
| Sertularidae g. sp. | * | * |  | * |
| *Spinimuricea klavareni* | * |  |  |  |
| *Villogorgia bebrycoides* |  | * |  |  |
| *Virgularia mirabilis* |  |  |  | * |
| **POLYCHAETA (4)** |  |  |  |  |
| *Filograna* spp. | * | * |  | * |
| *Myxicola infundibulum* |  |  |  | * |
| *Protula* sp. | * | * | * | * |
| *Sabella pavonina* | * | * |  |  |
| **ECHIURIDA (1)** |  |  |  |  |
| *Bonellia viridis* | * |  |  | * |
| **MOLLUSCA (13)** |  |  |  |  |
| *Bolinus brandaris* | * |  |  |  |
| *Calliostoma* sp. |  |  | * |  |
| *Caronia lampa* |  |  |  | * |
| *Chromodoris luteorosa* | * |  |  |  |
| *Flabellina affinis* |  | * |  |  |
| *Janolus cristatus* | * |  |  |  |
| *Neosimnia spelta* |  | * | * | * |
| *Marionia blainvillea* |  | * |  | * |
| *Loligo vulgaris* | * |  |  |  |
| *Pecten jacobaeus* |  |  |  | * |
| *Peltodoris atromaculata* | * |  |  |  |
| *Pteria hirundo* |  | * |  |  |
| *Solenogastres* g.sp. |  | * |  |  |
| **CRUSTACEA (8)** |  |  |  |  |
| *Ethusa mascarone* | * |  |  |  |
| *Galathea* sp. | * |  |  |  |
| *Macropodia* sp. | * |  |  |  |
| *Maja squinado* |  |  |  | * |
| *Munida* sp. | * |  |  | * |
| *Palinurus elephas* | * | * | * | * |
| *Parthenope* sp. |  |  |  | * |
| *Scyllarus* sp. | * |  |  |  |
| **BRYOZOA (7)** |  |  |  |  |
| *Adeonella calveti* | * | * | * | *** |
| *Hornera frondiculata* |  |  |  | *** |
| *Myriapora truncata* | * |  |  |  |
| *Pentapora fascialis* |  |  |  | *** |
| *Retiporella* sp. | ** |  |  | *** |
| *Schizobrachiella* sp. | * |  |  | *** |
| *Turbicellepora avicularis* | ** |  |  | ***** |
| **ECHINODERMATA (8)** |  |  |  |  |
| *Antedon mediterranea* | * | * | * |  |
| *Astrospartus mediterraneus* | * | * |  |  |
| *Cidaridae* g.sp. | * | * | * | * |
| *Echinaster sepositus* | * | * | * | * |
| *Eustichopus regalis* | * |  |  |  |
| *Holoturia polii* | * |  |  |  |
| *Holoturia tubulosa* | * |  |  | * |
| *Ophiotrix* spp. | * | * | * | * |
| **TUNICATA (6)** |  |  |  |  |
| *Aplydium tabarquensis* |  |  |  | * |
| *Clavelina lepadiformis* | * |  |  |  |
| *Clavelina nana* | * |  |  |  |
| *Halocynthia papillosa* | * |  |  | * |
| *Microcosmus* sp. |  |  |  | * |
| *Rhopalea* sp. | * |  |  |  |
| **OSTEICHTHYES (18)** |  |  |  |  |
| *Anthias anthias* | * | * | * | * |
| *Callanthias ruber* | * | * | * | * |
| *Cepola macrophthalma* | ** |  |  |  |
| *Conger conger* |  | * |  |  |
| *Coris julis* | * |  |  |  |
| *Helicolenus dactylopterus* |  | * |  |  |
| *Labrus mixtus* |  | * |  |  |
| *Lappanella fasciata* | * | * | * | * |
| *Macroramphosus scolopax* |  | * | * |  |
| *Muraena helena* |  |  | * | * |
| *Pagrus pagrus* |  | * |  | *** |
| *Phycis phycis* | * |  |  |  |
| *Scorpaena scrofa* | * | * | * | *** |
| *Serranus cabrilla* | * |  | * | * |
| *Thorogobius ephyppiatus* | * | * |  |  |
| *Trigla* sp. |  |  | * |  |
| *Trysopterus minutus* |  |  | * |  |
| *Zeus faber* |  | * | * |  |
| **CONDROICHTHYES (1)** |  |  |  |  |
| *Scyliorhinus* *stellaris* | * |  |  | * |

Table 1. Symbol legend: * = rare, ** = common, *** = very abundant
