# Supplementary material for: Deep Coral Oases in the South Tyrrhenian Sea
Source: PLoS One. 2012 Nov 21;7(11):e49870. doi: 10.1371/journal.pone.0049870 (PMC3503811; doi:10.1371/journal.pone.0049870)
Supplement: Table S2 — Results of the PERMANOVA testing for the effects of area on the abundance, taxa richness and composition of the target soft bottom corals. (DOC) [file pone.0049870.s004.doc]

**Table S2.** Results of the PERMANOVA testing for the effects of area on the abundance, taxa richness and composition of the target soft bottom corals. df = degree of freedom; MS = mean square; P = probability level;*** = P<0.001; ** = P< 0.01; * = P < 0.01; ns = not significant. Reported are also the pairwise comparisons.

| **Variable** | **Source** | **df** | **MS** | **Pseudo-F** | **P(MC)** | **Pairwise test** |
| --- | --- | --- | --- | --- | --- | --- |
| Abundance | Area | 3 | 0.004 | 4.09 | ** | S2 > S4 > S1 = S3 |
|  | Residual | 36 | 0.001 |  |  |  |
| Taxa richness | Area | 3 | 6.158 | 3.44 | * | S5 > Others |
|  | Residual | 36 | 1.792 |  |  |  |
| Assemblage | Area | 3 | 4239.3 | 3.15 | ** | n/a |
|  | Residual | 36 | 1346.2 |  |  |  |

Community composition

| **Contrast** | **t** | **P** |
| --- | --- | --- |
| S1 vs. S2 | 1.75 | ns |
| S1 vs. S3 | 1.27 | ns |
| S1 vs. S4 | 1.78 | * |
| S2 vs. S3 | 0.39 | ns |
| S2 vs. S4 | 2.86 | *** |
| S3 vs. S4 | 2.03 | ** |
